# Predictors of cervical cancer screening service utilization among commercial sex workers in Northwest Ethiopia: a case-control study

**DOI:** 10.1186/s12905-019-0862-7

**Published:** 2019-12-16

**Authors:** Bekele Atinafu Muluneh, Desta Debalkie Atnafu, Belaynew Wassie

**Affiliations:** 1 Family Guidance Association of Ethiopia, Bahir Dar, Ethiopia; 2 Department of Health System and Health Economics, School of Public Health, College of Medicine & Health Science, Bahir Dar University, Bahir Dar, Ethiopia; 3 Health System Management and Policy in School of Public health, Bahir Dar University, Bahir Dar, Ethiopia; 4 Department of Epidemiology and Biostatistics, School of Public Health, College of Medicine and Health Sciences, Bahir Dar University, Bahir Dar, Ethiopia

**Keywords:** Cervical cancer, Predictors, Screening utilization, Bahir Dar city, Ethiopia

## Abstract

**Background:**

Although an opportunistic approach of cervical cancer screening strategy had been implemented in Ethiopia, utilization of screening services among women is still low, accounted < 1%. We hypothesize that commercial sex women in Ethiopia faced a number of obstacles in order to access screening services. Identifying the predictors influencing utilizations of the screening services is an essential effort to tailor screening program towards increasing the utilization.

**Methods:**

An unmatched case-control study was implemented with a total sample size of 230 (46 cases and 184 controls). The study was conducted among commercial sex workers who attended confidential clinic opened for sex workers. Simple random sampling was employed. After the data were checked for completeness, consistency and accuracy, it was entered in to Epi nfo version 7 then exported to SPSS for further statistical analysis. Descriptive statistics were used to describe the profile of study participants. Logistic regression was employed to identify the predictors of cervical cancer screening uptake. *P* < 0.05 was computed to determine the level of statistical significance.

**Results:**

Cervical cancer screening utilization was associated with providers’ recommendation (AOR = 6.8; 95% CI: 2.3, 9.7), history of sexually transmitted infection (AOR = 6.9; 95% CI: 1.29, 7.2), frequency of facility visit (AOR = 4.8; 95% CI: 1.97, 11.8) and history of vaginal examination (AOR = 0.21; 95% CI: 0.1, 0.68).

**Conclusions:**

The level of cervical cancer screening service utilization was higher among women with history of STI, frequency of facility visit and providers’ recommendation. The level of cervical cancer screening service utilization was lower in women with previous vaginal examination.

## Background

Cervical cancer (CC) is the most deadly diseases of women primarily associated with chronic infection of the oncogenic types of human papilloma virus (HPV). CC, which is a non-communicable and preventable one, creates devastating effect in women of reproductive age groups. Globally, the incidence rate of cervical cancer was projected to be 500,000 and more than 150, 000 deaths were registered by the year 2012 [1–3]. About 90% of new cases among women were found in third world countries where utilization of the screening services was minimal [1, 2, 4]. In Sub-Saharan Africa (SSA), the incidence rate per 100,000 women was determined to be 25.2% where as in Ethiopia it has reached to 17.3% [5]. In Ethiopia, more than 27 million [6] and in Kenya about 10.32 million [7] of women in the reproductive age group were at risk of harboring HPV and contracting cervical cancer given the absence of integrated and organized screening service. A significant amount of the deaths had been occurred in resource limited countries including Ethiopia. Cervical cancer is the second leading causes of deaths among all cancer patients in Ethiopia which was estimated to be 16.3% per 100,000 females per annum [6]. The mortality rate of 20.3% in Nigeria [8] and 23.2% in SSA [9] were also accustomed to cervical cancer infections.

Cervical cancer is the most common sexually transmitted infection caused by HPV [10]. In women associated with commercial sex, the risk of HPV infection is highly prevalent mainly because of having multiple sexual partners, inconsistent condom use and co-morbidity with HIV/ AIDS [11]. The probability of development of HPV infection among sex workers was more than two times greater when compared to women from the general population [12]. About 23.7% of reproductive aged women, who developed cervical cancer, had been related to sexual practice [13].

Cervical cancer screening, which is safe, simple and reasonably low priced methods; has known to prevent cancer of apparently healthy people through early detection and treatment of any changes shown in the neck of the womb [3]. Therefore, screening for cervical cancer was the most effective strategy to detect and manage cases early particularly in resource limited countries with low level of advanced healthcare technologies. Despite higher burden of cervical case and the fact that easily preventable, the coverage of the screening program in highly affected women of the developing countries remained 5%. It was very low when compared to first world countries [14]. For example; in Nigeria, Ethiopia and Kenya, the rate of screening program coverage was highly exacerbated to be below 5.3, 1 and 3.2% respectively [5, 7, 15]. This made the problem very hard for those nations that could not initiate the simplest opportunistic screening services. However, in Ethiopia in most cities including Bahir Dar; a small-scale cervical cancer screening and treatment program was initiated with an integration of HIV/AIDS and reproductive health programs [2]. In Bahir Dar city, the service was also available for commercial sex workers free of any charges.

Among commercial sex workers, the predictors for cervical cancer screening service utilization were still not well described, particularly in a resource-limited setting, including Ethiopia. Predictors for cervical cancer screening uptake among sex workers of reproductive age group vary from one setting to another. Therefore, a context-specific study is mandatory for high risk segment of population where there is no universal HPV vaccine coverage. This study aims to identify predictors for cervical cancer screening service utilization in Northwest Ethiopia.

## Methods

### Study design and period

We conducted unmatched case- control study between October 05 and November 15, 2017 in Bahir Dar City, North West Ethiopia. Data was collected on commercial sex workers attending sex workers’ confidential clinic, Family Guidance Association of Ethiopia (FGAE), Bahir Dar Center. The data were collected from sex workers visited the clinic between the time period March to December 31, 2016.

### Study setting and participants

Bahir Dar City, where the study conducted, is the capital city of Amhra National Regional Government. It is found 565 Kms away from Addis Ababa, capital city of Ethiopia. According to 2016 national catch-up campaign for HIV testing and treating program, about 4725 sex workers were found in Bahir Dar city [16]. The total number of sex workers visited sex workers’ confidential clinic for any of the reasons between March 2016 to December 31st 2016 was 467. All were offered for cervical cancer screening service. Among them only 62 were accepted and screened for cervical cancer. Thus, in this case the prevalence of cervical cancer screening service uptake was determined to be 13.28%. The remaining were offered but declined from getting the services. Cases were sex workers who offered then accepted and ever had screened for cervical cancer and registered in Family Guidance Association Clinic for the period March 2016 to December 2016. Controls were those sex workers offered but declined from cervical cancer screening test. The screening of cervical cancer was conducted using either VIA or Pap smear test.

### Sample size determination & sampling method

The sample size was determined using Epi Info software Version 7 based on the double proportion formula. The study was planned to have 80% statistical power with 95% confidence interval and a case to control ratio of 1:4. Taking an assumption of the proportion of unfavorable attitude was 84.5% for the case and 98% for the control [17]; the estimated sample size was 42 for cases and 167 for controls. Allowing for a non-response rate of 10%, the final sample size was 230 (46 cases and 184 controls). The sample size was determined for exposure status of different variables. However, the largest sample size among those exposure variables was taken. Simple random sampling was employed to recruit study participants.

### Data collection tool, technique and procedure

A structured and pre-tested questionnaire adapted to the local context was used to collect data through face-to-face interview. The tool validity and reliability was checked using Cronbach’s alpha, value was 0.81. Trained nurses who were in charge of clinical setups conducted face- to- face interviews with study participants by using structured questionnaires. The questionnaire was first prepared in English and later was translated into Amharic (local language) and back to English to keep its consistency. The data were collected to assess socio-demographic factors, enabling factors and needs factors.

Study participants (cases and controls), who ever had invited for the screening in the clinic and met the inclusion criteria, were selected using simple random sampling technique after all the charts of sex workers and cervical cancer screening logbook has been retrieved. After the cases and controls have been selected, women were called on to the center for interview using their phone address collected from the chart.

### Data quality management

To ensure the quality of data, training about data collection procedures was provided for both data collectors and supervisors. Nurses who were in charge of clinical setups conducted the data collection process. Pre-testing of the data collection tool was also carried out among 5 cases and 20 controls in clinics that were not included in the current study. The investigators and supervisors closely checked the data collection procedures on the spot. Any questionnaire with a defect was rejected and counted as a non-response.

### Data processing and analysis

After the data collection tools coded, data were checked and entered in to the computer using Epi Info version 7 software. Data were exported to SPSS version 20 for cleaning, editing, checking for missing values and further analysis. Frequencies and percentages were used to describe the profile of study participants. To compare predictors of cervical cancer screening service utilization between cases and controls, X^2^ was computed. In addition, based on Hosmer-Lemeshow applied logistic regression guide, variables with a *p*-value *<* 0.2 in the binary logistic regression analysis were fit in to multivariable regression analysis and *p-value < 0.05* was considered to identify significant independent predictors for cervical cancer screening service utilization. According to Hosmer-Lemeshow goodness of fit test, the model was fitted to the data.

Respondents’ knowledge and attitude about cervical cancer screening was measured using 13 point knowledge score and 10 point attitude score questions that carried a total of 44 correct responses for knowledge and 50 for attitude. Each correct responses was given a score of 1 and wrong responses a score of 0. The questionnaires included risk factors, symptoms, prevention, susceptibility, severity, screening practice and treatment of the disease etc. Sex workers with total score greater than or equal to the mean value were categorized as having “sufficient knowledge” or “favorable attitude” and those with score less than the mean were categorized as having “insufficient knowledge” or “unfavorable attitude” [17].

### Ethical considerations

Ethical approval was obtained from Amhara Public Health Institute ethical committee (reference number: APHI/01/742). Written permission to conduct the study was obtained from Family Guidance Association of Ethiopia. Written informed consent was obtained from each study participants. After the interview had conducted, participants with cervical cancer or precancerous lesion were linked to cervical cancer treatment centre for further treatment. The name of respondents was not written on the questionnaire and all information obtained from the health institution & respondents were kept confidential.

## Results

The overall response rate was determined to be 95.22%. The response rate for cases was 100%, whilst for controls it was 94.02%.

### Predisposing characteristics of commercial sex workers

The mean age of the cases was 29.5 (±4.4) years, and that for controls was 25.8 (±3.9) years. The difference among the means was also statistically significant. A substantial proportion of respondents were found in the age group below 30 years, 67.4% among cases and 91.9% among controls. Comparison of predisposing factors between cases and controls was made using X^2^ test; there was no significant difference in the variable interest of educational background, religion and ethnicity. However, there was a statistically significant difference in the remaining variables. Of the total respondents, 37% cases and 57.3% controls had sufficient knowledge about cervical cancer and screening (Table 1).

**Table 1:** Socio-demographic and related characteristics of commercial sex workers in Bahir Dar city, between March 2016 to December 31st, 2016

Variable		Cases(Screened)	Controls(Not screened)	Difference	X^2^	*p*-value
		f (%)	f (%)			
Age group (year)	Mean; Standard deviation	μ ± δ = 29.5 ± 4.4	μ ± δ = 25.8 ± 3.9	3.7 ± 0.5	−3.56*	0.001
	< 30 years	13(28.3)	96(55.5)	7.2	10.78	0.001
	≥30 years	33(71.7)	77(44.5)	27.2		
Educational status	Unable to read and write	24(52.2)	71(41)	11.2	2.78	0.43
	Primary education	16(34.8)	67(38.7)	−3.9		
	Secondary education	3(6.5)	24(13.9)	−7.4		
	Collage and above	3(6.5)	11(6.4)	0.1		
Religion	Orthodox	45(97.8)	168(97.1)	0.7	0.07	0.792
	Muslim	1(2.2)	5(2.9)	0.7		
Ethnicity	Amhara	40(87)	150(86.7)	0.3	0.002	0.96
	Others	6(13)	23(13.3)	−0.3		
Duration in working as sex workers	< 6 years	23(50)	117(67.6)	−17.6	4.89	0.027
	≥6 years	23(50)	56(32.4)	17.6		
Knowledge about CC and its screening	Sufficient knowledge	17(37)	99(57.2)	−20.8	5.993	0.014
	Insufficient knowledge	29(63)	74(42.8)	20.2		
Attitude about CC and its screening	Favorable	35(76.1)	65(37.6)	38.5	21.725	0.001
	Unfavorable	11(23.9)	108(62.4)	−38.5		
Provider’s recommendation	Yes	19(41.3)	150(86.7)		42.513	0.001
	No	27(58.7)	23(13.3)			
Source of information	Healthcare workers	25(54.3)	55(31.8)		3.907	0.048
	Mass media	2(4.3)	10(5.8)			
	Friends	19(41.3)	108(62.4)			
Women perception about convenient time for screening	Yes	2(4.3)	33(19.1)	−14.8	5.87	0.015
	No	44(95.7)	140(80.9)	14.8		

### Enabling characteristics of commercial sex workers

The average monthly income of the sex workers over all was Ethiopian Birr (ETB) 1948.86 ± 922.072, 27.4 ETB was equivalent to 1 US$. There was no significance difference in their income in the likelihood of up taking cervical cancer screening service. However, history of health institution attendance and consistent condom use did vary significantly with sex workers’ cervical cancer screening service uptake status (Table 2).

**Table 2:** Respondents’ income and healthcare access related factors in Bahir Dar City, between March 2016 to December 31st, 2016

Variables		Cases(Screened)	Controls(Not screened)	Difference	X^2^	*p*-value
		f (%)	f (%)			
Income per month / ETB	< 700	4(8.7)	11(6.4)	2.3	3.39	0.184
	700–2000	33(71.7)	104(60.1)	11.6		
	> 2000	9(19.6)	58(33.5)	−13.9		
History of consistence condom use	Yes	17(37)	99(57.2)	−20.2	5.993	0.014
	No	29(63)	74(42.8)	20.2		
Frequency of facility visit/year	< 6 years	31(67.4)	34(19.7)	47.7	39.678	0.001
	≥ 6 years	15(32.6)	139(80.3)	−47.7

**Table 3:** Health conditions and perceived healthcare needs characteristics of respondents’ in Bahir Dar City, between March 2016 to December 31st, 2016

Variables		Cases(Screened)	Controls(Not screened)	Difference	X^2^	*p*-value
		N (%)	N (%)			
History of abnormal vaginal discharge	Yes	8(17.4)	17(9.8)	7.6	2.056	0.152
	No	38(82.6)	156(90.2)	−7.6		
History of abortion	Yes	8(17.4)	12(6.9)	10.5	4.764	0.029
	No	38(82.6)	161(93.1)	−10.5		
History of vaginal examination	Yes	39(84.8)	103(59.5)	25.3	10.158	0.001
	No	7(15.2)	70(40.5)	−15.3		
History of STI	Yes	2(4.3)	49(28.3)		11.693	0.001
	No	44(95.7)	124(71.7)

**Table 4:** Barriers to cervical cancer screening service utilization among respondents in Bahir Dar City, between March 2016 to December 31st, 2016 (*N* = 173)

Variables	Frequency (*f*)	Percentage (%)
Fear of test result (perceived susceptibility) (*N* = 173)	34	19.65
Lack of provider recommendation (*N* = 173)	23	13.29
Lack of female screener (provider) (*N* = 173)	3	1.73
Shortage of time (being busy) (*N* = 173)	20	11.56
Being healthy (not at risk) (*N* = 173)	122	70.52
Lack of convenient clinic time (N = 173)	140	80.92

**Table 5:** Binary and multivariable analysis to identify predictors of cervical cancer screening service utilization by respondents in Bahir Dar City, between March 2016 to December 31st, 2016

Variables		CC Screening Service Utilization		COR (95%CI)	AOR (95%CI)	*p*-value
		Yes	No			
Age group (years)	< 30	31	159	1	1	
	> 30	15	14	0.18(0.08, 0.42)	0.09(0.025,0.4)	0.98
Consistence condom use	No	29	74	1		
	Yes	17	99	2.28(1.17,4.46)	0.5(0.19,1.38)	0.19
Abnormal vaginal discharge	No	38	156	1	1	
	Yes	8	17	1.93(0.78,4.81)	0.98(0.2,4.92)	0.98
Attitude towards CC and its screening	Favorable	35	65	5.29(2.5,11.13)	2.62(0.93,7.33)	0.068
	Unfavorable	11	108	1	1	
Provider’s recommendation	No	27	23	1	1	
	Yes	19	150	0.11(0.05,0.23)	6.76(2.3,9.7)	0.002*
Duration in working as sex workers	< 6 years	23	117	1		
	> 6 years	23	56	2.09(1.08,4.04)	1.02(0.4, 2.5)	0.606
History of abortion	No	38	161	1		
	Yes	8	12	0.35 (0.14, 0.9)	0.43(0.12, 1.5)	0.186
History of vaginal examination	No	7	70	1	1	
	Yes	39	103	0.26(0.11, 0.62)	0.21(0.1,0.68)	0.009*
History of STIs	No	44	124	1	1	
	Yes	2	49	8.7(2.03, 9.25)	6.92(1.29,7.2)	0.024**
Knowledge about CC & screening.	Insufficient knowledge	29	74	1	1	
	Sufficient Knowledge	17	99	2.3(1.17, 4.46)	0.99(0.4, 2.44)	0.988
Frequency of facility Visit /year	< 6	31	34	1	1	
	> 6	15	139	8.4(4.11, 17.39)	4.82(1.97,11.8)	0.001*

### Health conditions and perceived healthcare needs characteristics of commercial sex workers

Comparison of perceived healthcare needs characteristics between cases and their controls were made using x^2^ test. There were significant differences in the distributions of the sex workers by history of vaginal examination, history of abortion, and history of sexually transmitted infection (STI) categories among cases and controls. A tremendous proportion of respondents among cases (82.6%) and controls (90.2%) were found with respect to no history of abnormal vaginal discharge. There was also no statistical difference between cases and controls with regard to this variable (Table 3).

### Reasons and barriers for acceptance of cervical cancer screening service

About 80.3% of commercial sex workers, who had history of facility visiting greater than six times per annum, were failed to have Pap smear cervical cancer screening service. The primary reasons for them never having cervical cancer screening were depicted in (Table 4) below.

### Predictors of cervical cancer screening service utilization

Comparison of variables that were statistically significant with cervical cancer screening service utilization on crude analysis was adjusted for possible confounders as shown in Table 5. Thus, a number of variables remained in the multivariable model: providers’ recommendation, history of sexually transmitted infection and history of facility visiting were independent predictors for increased cervical cancer screening service utilization. However, history of vaginal examination had an independent protective effect against cervical cancer screening service utilization. Sexually transmitted infections (STI) increases utilization of cervical cancer screening service, the odds of have been screened for cervical cancer was higher among study participants who had history of STI than those who did not diagnosed and treated for STI. In other words, most commercial sex workers (cases) were diagnosed and treated for STI as compared to their controls and this difference was statistically significant (AOR = 6.92; 95% CI:1.29,7.2). The odds of cervical cancer screening service utilization were 6.76 fold higher in commercial sex workers who had been recommended by healthcare professionals for up taking the service (AOR = 6.76; 95% CI: 2.3, 9.7). Commercial sex workers with frequency of healthcare facility visiting greater or equal to 6 times per year had 4.8 fold higher utilization of cervical cancer screening services than the counterpart (AOR = 4.8; 95% CI: 1.97, 11.8) whereas, utilization of cervical cancer screening services was 79% lower among sex workers having prior and repeated vaginal examination (AOR = 0.21; 95% CI: 0.1, 0.68). And the remaining variables did not show significant association in the multivariable model.

## Discussion

This study has included commercial sex workers having a high response rate. This might be due to implementation of voluntary basis informed consent and appropriate arrangements taken by the local authorities. The high rate of response could minimize the differences that might exist between the sample and population parameter. To the level of our knowledge concerned, this is the first study in its kind to identify predictors of cervical cancer screening service utilization among commercial sex workers in Bahir Dar city, North West Ethiopia. The occurrence of new cervical cancer cases in Ethiopia among sex workers had increased rapidly when compared to the general population. This rapid occurrence was mainly attributed to factors include: multiple sexual practice, early sexual debut, history of sexual transmitted diseases infection including HIV/AIDS, sexual exposure to someone with cervical neoplasia and long term contraceptive use etc. [6, 9, 10, 17, 18]. To mitigate the health impact of cervical cancer, targeting on barriers for screening among commercial sex workers, where their early cervical cancer screening practice was low, is mandatory.

The mean age of the cases was 29.5 years while the mean age of controls was 25.8 years. The cases were about 4 years older than the controls and this difference is also statistically significant (*P* < 0.001). Women whose ages 30 years and above are recommended to had screening test for cervical cancer [2]; 32.6% of sex workers whose mean age greater than by 4 year had ever screened for cervical cancer whereas 8.1% of women whose mean age less than by 4 year had not screened.

Education is a backbone and precondition to developing awareness about healthcare services. However, females in many resource limited countries are lack of access to education for healthcare information and this might worsen to promoting their health, including the practice of screening. Despite this truth, the study affirmed that educational background was not found in the multivariable regression model and just not been a predictor of cervical cancer screening service utilization, similar to previous studies [14, 18, 19]. Thus, increasing formal education may not be necessarily successful in bringing behavioral change towards cervical cancer prevention and control particular to in this segment of target population.

Furthermore, the level of knowledge on cervical cancer & screening was not an independent predictor for screening service utilization, in contrast to previous studies [14, 19, 20]. Nonetheless, a significant difference was shown in the level of knowledge between cases and controls (P < 0.05). Literatures showed that educating of women on cervical cancer improved their level of knowledge and brought an incremental effect on utilization of cervical cancer screening. However, education and level of knowledge in this segment of population had never associated with cervical cancer screening utilization. The probable explanation for the difference existed between the studies being compared might be the nature of population studied, and the role of healthcare providers. The populations found in the current study were a segment of women associated with commercial sex and believed to be risky for any disease entities. Hence, impreference to the general population; most healthcare providers would like to recommend them to take the screening service at first contact of any healthcare facility regardless of their knowledge characteristics. Respondents in Thailand and Jamaica mentioned above were general population groups while women in Addis Ababa had history of HIV/ ADIS [17, 19, 20].

A scholarly findings from different literatures showed that attitude towards susceptibility to cervical cancer had a profound independent effect on screening uptake, conversely reported by the current study [20–22]. This could perhaps account for the difference in acceptability of the screening program and cultural diversity found within study settings. As the level of perceptions of cervical cancer might vary from person to person, the same is true from country to country. The belief and perception on healthcare and medicine of communities where this study undertaken was different from Jamaica and others found in the world. This is also because of the fact that the functioning primary healthcare system in Ethiopian was not yet well integrated with existing beliefs and traditions of the community [23].

Cervical cancer is a relatively rare consequence of the most common sexually transmitted infections in the world and sex workers are at higher risk of acquiring cervical cancer infections because of their multiple sexual behavior and inconsistent condom use [2, 24, 25]. Thus, opportunistic cervical cancer screening service should be expanded in every clinic in order to increase screening service uptake. Sex workers, who had history of STI or HIV/AIDS, should be initiated and offered for cervical cancer screening during first clinic contact. Furthermore, we found that the odds of undergoing cervical cancer screening services among women, who had history of STI, were 6.9 times higher than those who didn’t have any infections related to sexual practice, which corroborates the findings of other studies conducted in Turkey [26], Mekelle [27], Botswana [28], Zambia [29] and Ethiopia [27, 30]. This might be the trend that women who came for diagnosis and treatment of STI in healthcare facilities were offered for cervical cancer Pap test. It could signify the presence of links between services with in facility and was considered an important step towards prevention and controlling of cervical cancer.

Sex workers, who visited health institution frequently, would have higher chance of getting comprehensive information about early cervical cancer screening. As a result, commercial sex workers particularly those HIV/ ADIS victims should be encouraged to attend health providers regularly in order to check for cervical cancer. In this study, frequency of facility visit by sex workers had positive effect on cervical cancer screening uptake. This was consistent with previous study conducted in Jamaica [20]. In Ethiopia, cervical cancer screening services were strategized at small scale level being integrated with management of HIV infection and reproductive health. Reports in Addis Ababa also showed that ART visits were the main reason for women having their first Pap test [17].

Healthcare facilities’ recommendation is an opportunity to counseling women by which the negative attitudes about cervical cancer and screening could be addressed. Women should be responsive to their healthcare providers who inform them about the characteristics and its preventive measures of cervical cancer. The current finding was also consistent with prior studies in that commercial sex workers, who had history of healthcare provider recommendations, were more likely to up taking cervical cancer screening [2, 31–34]. A study conducted in Thailand revealed that embarrassment and longer waiting time had a negative impact on the decisions of women to attend cervical cancer screening service [19]. Most women become uncomfortable with the idea of vaginal examination of private parts especially when Pap smear was collected by male providers [35]. We found that history of vaginal examination had an inverse association with uptake of cervical cancer screening, supported by study conducted in India, Jamaica and Thailand [19, 36, 37]. Conversely, studies conducted in Nigeria [38] and Turkey [26] showed that history of vaginal examination were positively correlated with cervical cancer screening uptake. The possible reason for this discrepancy would be due to the difference in protocol of case management and administration (painful procedure of vaginal examination), allocation of female providers, healthcare resource allocation, unfriendly and poor counseling practice. Thus, the health system on this scenario should standardize the existing clinical procedure assisted by availability of adequate resources.

### Limitations

We used a case–control study; which lacked a clear pattern of causality between predictors and outcome variables that could be tested. Since our study was focusing on commercial sex workers attending healthcare facility, the results may not be generalizable to all targeted women of comparable age.

## Conclusions

Although attitude and knowledge about cervical cancer and its screening, availability of convenient clinic time, history of abortion, consistent condom use, source of information, age group, duration of work as commercial sex and other factors were correlated to cervical cancer screening service utilization; only history of STI, providers’ recommendation, frequency of healthcare institution visit and history of vaginal examination were independently predicting the level of cervical cancer screening service utilization. Hence, guidelines (strategy) that promote integration of the screening service to the routine care and treatment (opportunistic screening) should be developed and implemented targeting on marginalized and other key affected commercial sex workers. Assigning female cervical cancer screener and availing convenient clinic time should also be given due emphasis by the ministry and sector health offices.

## Data Availability

All relevant materials and data supporting the findings of this study are available without restriction. Contact to this e-mail: destad2a@gmail.com when needed.

## Abbreviations

AOR: Adjusted odds ratio
ART: Anti retro-viral therapy
CCSU: Cervical cancer screening utilization
CI: Confidence interval
ETB: Ethiopian birr
FGAE: Family guidance association of Ethiopia
FSWCC: Female sex workers confidential clinic
HIV: Human immune deficiency virus
HPV: Human papiloma virus
SSA: Sub Saharan Africa
STI: Sexually transmitted illness
US$: United States of America dollar
VIA: Visual inspection with acetic acid
WHO: World health organization
